# An application of AHP and fuzzy entropy-TOPSIS methods to optimize upstream petroleum investment in representative African basins

**DOI:** 10.1038/s41598-024-57445-9

**Published:** 2024-03-23

**Authors:** Zhihua Cui, Olusoji Lawrence Taiwo, Peace Mawo Aaron

**Affiliations:** 1 School of Civil and Transportation Engineering, Ningbo University of Technology, Ningbo, China; 2 Department of Geology and Geophysics, University of Aberdeen, Aberdeen, UK; 3 Department of Earth Sciences, Durham University, Durham, UK

**Keywords:** Investment assessment, Upstream petroleum investment, African petroleum basins, Analytic hierarchy process, Fuzzy entropy-TOPSIS, Economic geology, Energy and society, Socioeconomic scenarios, Sustainability

## Abstract

The growing demand of China for petroleum heightens the complexities and prospects in worldwide investments, necessitating refined and strategic investment approaches. Evaluating the potential of different hydrocarbon-potential areas needs more comprehensive scientific evaluation models. This study aims to establish a Comprehensive Investment Potential of Petroleum (CIPP) framework for targeted sedimentary basins by using an integrated approach that combines the Analytic Hierarchy Process (AHP) and the Entropy-Weighted Fuzzy TOPSIS models. We focus particularly on representative African basins to inform strategic decision-making for the Chinese overseas petroleum enterprises. We firstly interpret the geological condition of these petroleum basins by researching multiple databases and proprietary research data. Then, we use a combined approach of ranking-classification-correlation analysis to evaluate 17 representative basins, taking into account both overall and individual key performance indicators. Our findings suggest the Illizi Basin and the Offshore Côte d'Ivoire Basin could be the most favorable for investment and development. Those like Southwest African Basin warrant cautious consideration. The new evaluation model and computational workflow offer an effective workflow for assessing multiple petroleum basins. This work provides not just practical investment strategies for companies aiming for African petroleum basins, but also a transferable methodology for optimizing investment decisions.

## Introduction

As the role of petroleum resources grows in the industrial production of China, the demand has also increased over the years^1,2^. Compensating for domestic petroleum production shortfalls makes international upstream investment and development increasingly imperative. Nevertheless, for Chinese enterprises, this investment arena is fraught with multifaceted challenges of considerable complexity^3–6^. Affected by various factors such as geography, economics and politics, optimizing investment strategies have become critically important^6^. Specifically in Africa, the abundance of petroleum resources has attracted investments and developmental efforts from various countries and regions, including China and Western nations^7^. However, there is currently a lack of comprehensive and scientific methodologies for evaluating the exploration and development potential of various African basins. This absence introduces significant uncertainties for similar investors and policymakers. The lack of targeted research and scientific ranking mechanisms often leads to hesitancy among various capital stakeholders when selecting target basins for expansion.

Previous research focus on the evaluation of petroleum resource utilization and associated investment returns^8^. These studies, using a variety of evaluation methodologies, predominantly concentrate on economic dimensions such as risk investment, price volatility and rates of return on capital^8–16^. However, very few studies offer classification and ranking for specific regional clusters under investigation^17^.

Some research endeavors provide investment risk assessments tailored for individual countries^18^. There are also commendable studies worth noting that focus on downstream refinery-oriented overseas strategic investment^19^. However, these works rarely adopt an all-sided approach to strategic investment risk evaluation by incorporating factors like petroleum geological conditions, development status and socio-political environment. While some research does consider geological conditions, these studies often exhibit a level of simplification in their indicators and dimensions that is inadequate for comprehensive analysis^20,21^.

The scarcity of basin-level offshore oil investment evaluations reflects the challenge of accounting for numerous interconnected factors. Compared to assessments at the national level, evaluations at the basin level can take into account more geological conditions related to oil and gas, as these factors are of significant importance. We suggest that, in order to better evaluate the degree of oil and gas investment, replacing the national level with the basin level can highlight the effectiveness of the investment. This approach allows for a greater focus on upstream oil and gas exploration, as the majority of oil and gas reserves remain undeveloped.

Besides, in the realm of upstream petroleum industry investment, some exploratory yet little research has been conducted using various evaluation algorithms within Decision-making Support Systems (DMS)^22^. In contrast, evaluative application research in other domains demonstrates a variety of models that exhibit superior evaluation and classification results when dealing with multi-objective scenarios under complex conditions^23–28^. The integration of subjective and objective judgments, employing complex algorithms such as Analytic Hierarchy Process (AHP), Technique for Order Preference by Similarity to Ideal Solution (TOPSIS), and Entropy methods, in evaluation techniques is extensively applied^27,28^. In the calculation of assessment indicator weights, we have considered a similar method that combines both subjective and objective factors, thereby enabling a comprehensive consideration of the indicator information of the assessment object in the most scientific manner. This model introduces a new method for assessing the overseas oil resource investment environment at the basin level.

The research gap can be identified in the assessment of the upstream oil investment potential of African basins. On one hand, previous studies or evaluation models lack consideration of the factors of oil resource utilization and seldom involve a comprehensive assessment of oil geological conditions, development status, and socio-political environments. Moreover, there is a lack of an optimized mathematical evaluation model for assessing, classifying and ranking all African oil and gas basins, with multidimensional comparisons both across and within dimensions. This presents significant uncertainty and a lack of a macro perspective for oil and gas investors when selecting target basins in the region.

Therefore, there is requirement for studies that concentrate on choosing oil resources from various geographical areas, as there is a noticeable absence of appropriate assessment frameworks specifically designed for this situation. A comprehensive consideration at the basin level, integrating both subjective and objective factors, allows investment strategies to focus more on the inherent potential of oil and gas reserves. The primary objective of this paper is to innovate upon existing comprehensive and complex evaluation models from other fields to assess the investment potential of targeted petroleum regions. This aims to deepen both the research and understanding of basin selection in the petroleum investment field, offering an advanced yet effective evaluation model as a new perspective for fellow researchers and practitioners. Specifically, this study seeks to refine the selection of representative basins in Africa for potential petroleum investment and development, providing a comprehensive evaluation framework for targeted basin clusters. This research can address the research gap by establishing a rational and effective mathematical evaluation model, filling the void of a lack of systematic, multi-level, and multi-dimensional rankings and classification systems for African oil and gas basins that can integrate the attributes of oil and gas resources with economic and social environmental factors.

To achieve this, the study leverages multiple databases and prior research materials to provide an application to evaluate the upstream petroleum investment within African basins: (1) utilize Interpretive Structural Modeling (ISM) to construct a multi-dimensional comprehensive indicator system, (2) use the Analytic Hierarchy Process (AHP) based on expert scoring and the entropy method to balance subjective and objective evaluations, thereby establishing the weights of various indicators, (3) apply an enhanced Entropy-Weighted Fuzzy TOPSIS method for comprehensive ranking and evaluation, (4) classify the evaluation results and (5) include a correlational analysis of internal key performance indicators to further enrich and validate the evaluation results.

## Material and analysis

### Study area

The African continent has a land area of approximately 3.02 × 10^9^ km^2^, which includes over 60 petroleum resources. As of the end of 2020, the proven oil reserves of Africa stood at 125.1 billion barrels, accounting for 7.2% of the world's total reserves. Simultaneously, the proven natural gas reserves were estimated at 12.9 trillion cubic meters, constituting approximately 7% of global reserves. Africa ranks third in terms of petroleum reserves.

Although Africa has abundant petroleum reserves, progress in the petroleum and natural gas sectors has been slow. This is due to historical factors, technological limitations and complex political situations. Currently, most exploration and development efforts are focused on the edges of the continent, mainly in the east and west coasts, where resources are highly concentrated. Despite low levels of activity and clear regional imbalances in petroleum exploration and development, the improving political situation in Africa is attracting many global oil companies to the region. This suggests that investment opportunities in African petroleum field are likely to increase.

### Data and qualitative analysis

In this study, we primarily rely on the most recently available data up to 2020 from the IHS and Tellus databases, as well as accumulated independent research materials, for our data sources. Specifically, we have carefully selected 17 petroleum basins in Africa that have high investment value as our research sample.

This study applies a multidisciplinary geological analysis framework that has various sub-disciplines including stratigraphy, sedimentology and structural geology to conduct a comprehensive analysis of the geological characteristics of the selected 17 petroleum basins. Additionally, we carried out an extensive review, data compilation, and analysis of historical and current exploration activities for each selected basin. This includes confirmed petroleum fields, ongoing exploration projects, as well as regions with future exploration potential. Moreover, we assessed the current development status of each basin, which covers existing development projects and related technological requirements, and conducted quantitative estimations of petroleum reserves based on available data. Beyond geological and engineering considerations, this study further involves a multi-dimensional analysis incorporating political, economic, and cultural factors. Finally, and significantly, we highlight that all indicators are considered to be within an acceptable range, in which they display a proportional relationship, implying that higher values signify either improved or deteriorated outcomes accordingly.

### Interpretation and quantitative evaluation

We conducted in-depth interactive survey questionnaires and focused discussions. Based on the compilation and summary of the data and materials, we first carried out a detailed interpretation of the petroleum basin clusters. This deconstruction work was conducted based on multiple factors such as geographical location, petroleum reserves, development difficulty and political stability, aiming to facilitate a more systematic understanding of the characteristics of each basin. Then, upon completing the geological analysis, we employed a standardized scoring system to quantitatively evaluate each basin in terms of stratigraphy, sedimentology and structural geology. This evaluation system was collaboratively developed and aims to provide an objective and comparable method for assessing the geological conditions of each basin. Finally, we collected and organized a large volume of raw data, including information on petroleum reserves, historical records of exploration activities and technological requirements from sources above. This data was carefully sorted and organised for use in the following statistical and computational models, for providing a more precise quantitative assessment of petroleum reserves and development potential.

### Classifying indicators

We have exhaustively listed all important indicators and formed a comprehensive set of indicators, establishing classifications between subjective and objective indicators. As shown in Table 1, for modeling purposes, these subjective indicators are marked as *S*, while the objective ones are labeled as *S&R*. We generated subjective evaluation values (1–10) for all indicators. For some indicators with precise objective evaluation values, these subjective values will be combined through subsequent work to ensure the comprehensiveness and depth of this study on evaluation modeling. The principle of classifying these subjective and objective factors is mainly based on whether they have precise numerical data, derived from multiple accessible databases.

**Table 1:** Weighting indices across three levels: L0, L1, L2 and L3. In L3, a plus sign (+) indicates higher numerical values are more optimal, whereas a minus sign (−) means less optimal. S&O refers to indicators that considers both subjective and objective factors, whereas S specifically refers to subjective considerations only.

L0	L1		L2		L3	
Comprehensive investment potential of petroleum	Exploration status	0.407	Fundamental geological petroleum condition	0.069	Source rock condition (+; S)	0.024
					Reservoir condition (+; S)	0.021
					Cap rock and preservation condition (+; S)	0.017
					Petroleum migration pathways (+; S)	0.018
					Petroleum accumulation environment (+; S)	0.014
			Petroleum resource potential	0.284	Proven reserves (MMboe) (+; S&O)	0.099
					Producible reserves (MMboe) (+; S&O)	0.094
					Potential reserves (+; S)	0.013
			Exploration progress	0.314	Exploration well density (km^2^/well) (+; S&O)	0.109
					Exploration success rate (+; S&O)	0.029
					Exploration maturity (+; S)	0.020
					Existing seismic data (+; S&O)	0.062
					Estimated subsequent exploration investment (+; S)	0.011
	Development and production	0.286	Development condition	0.049	Development investment return rate (+; S)	0.017
					Extraction technology (+; S)	0.015
					Development cost per well (bbl/d) (−; S&O)	0.033
					Development well cycle (days) (−; S&O)	0.024
					Development well production (bpd) (+; S&O)	0.036
					Development difficulty (−; S)	0.032
			Production condition	0.158	Production rate per well (bbl/d) (+; S&O)	0.055
					Overall production efficiency (+; S)	0.028
					Recovery rate (%) (+; S&O)	0.025
					Extraction cost per well ($/bbl) (−; S&O)	0.031
	Local environment	0.307	Environment for petroleum resource utilization	0.052	Port transportation condition (+; S)	0.018
					Infrastructure (+; S)	0.025
					Petroleum transportation condition (+; S)	0.027
					Local market demand (+; S)	0.016
					Ecological impact (+; S)	0.010
			Investment environment	0.073	Social stability (+; S)	0.026
					Political security (+; S)	0.019
					Local policies and regulations (+; S)	0.016
					Market openness (+; S)	0.015

## Preparation and methods

**Figure 1:**
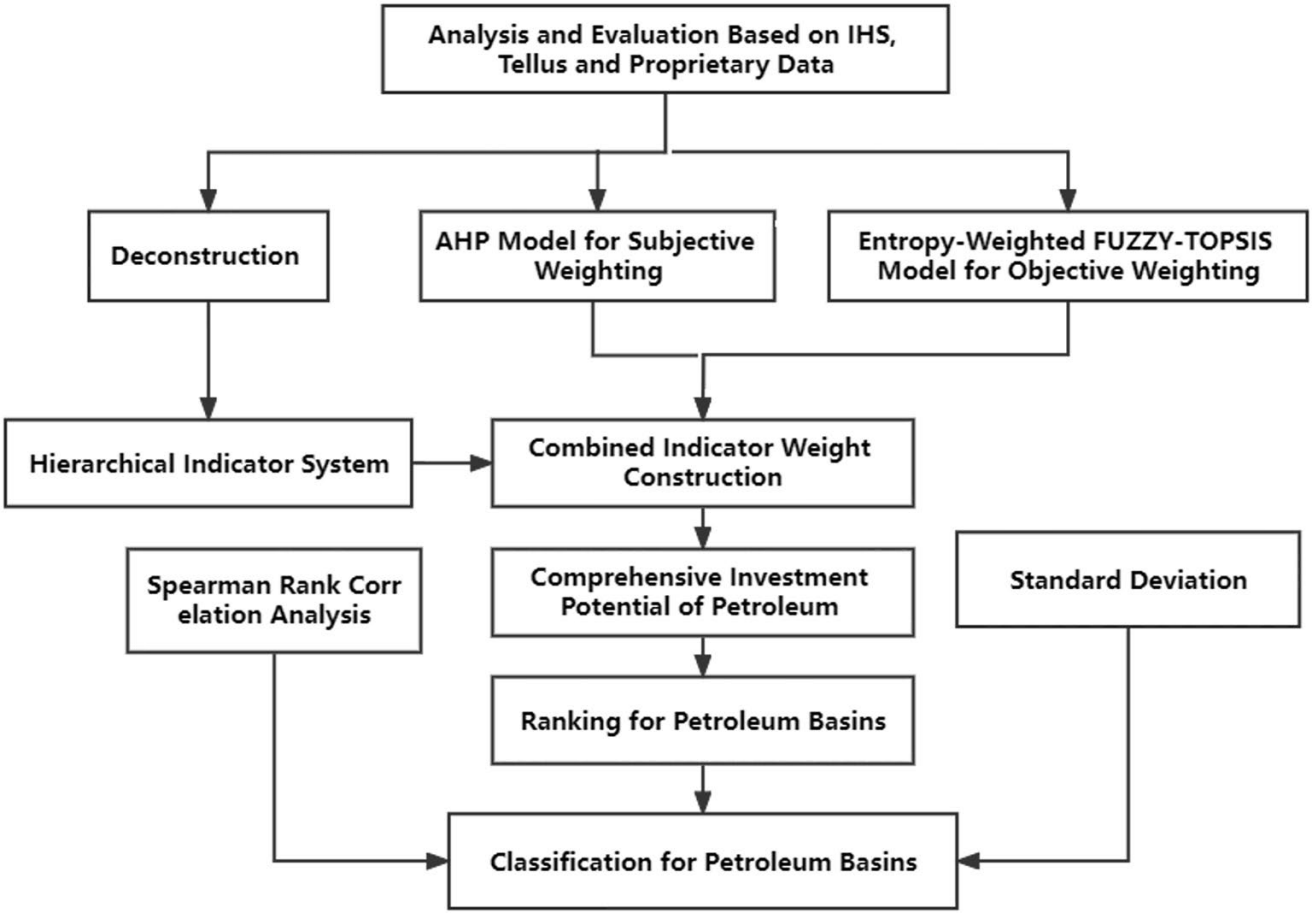
Workflow diagram of the whole evaluation process.

To comprehensively assess the investment potential in petroleum resources for the 17 representative basins in Africa, this study developed an integrated evaluation model. The model combines three algorithmic methods: Analytic Hierarchy Process (AHP), Entropy Method and Technique for Order of Preference by Similarity to Ideal Solution (TOPSIS), forming a comprehensive evaluation workflow (Fig. 1). This evaluation system takes into account both subjective factors like expert opinions and objective indicators to provide a more accurate quantification of the investment value for each basin.

### Modeling strategy

This study utilizes a multi-dimensional, multi-faceted comprehensive indicator system for evaluation. For international petroleum companies, the assessment of resource potential has considerations of petroleum reserves, exploration status, as well as the availability of local infrastructure support, as indicated in related research^29^.

Following in-depth discussions by our panel of experts, we identified three primary indicators: exploration status, development and production capabilities and local environmental conditions. To further refine these primary indicators, we applied Interpretive Structural Modeling (ISM) for optimization. The ISM method is capable of decomposing a complex system into multiple key elements and revealing the inherent logical and structural relationships among these elements through matrix operations and topological analysis^17,30^. This model offers decision-making support in an intuitive and dynamic manner and excels in comprehensiveness, flexibility and scientific validity. Using this method enables a more comprehensive quantification and comparison of the resource utilization potential across various petroleum basins in Africa.

### Methods

In this study, we address a complex multi-objective ranking problem, assessing the investment potential in upstream petroleum in African basins across various dimensions. Given the complexity and multidimensionality of this issue, we opted for a combined approach of the Analytic Hierarchy Process (AHP) and the Entropy-Weighted Fuzzy TOPSIS method, aiming to introduce an innovative evaluation framework to the oil and gas investment decision-making domain^31,32^. The choice of this methodology is grounded in its proven effectiveness in handling complex decision-making scenarios across diverse fields such as engineering, management and environmental science. AHP facilitates the clarification of the decision-making structure within a multi-tiered framework, allowing for the determination of relative importance of different factors through expert scoring. Meanwhile, the Entropy-Weighted Fuzzy TOPSIS method leverages objective data to manage uncertainty and fuzziness, thus integrating expert judgment with data objectivity in the final ranking. The advantage of this integrated methodological approach in our research lies in its ability not only to enhance the accuracy and reliability of evaluations but also to provide a fresh perspective and tool for research and practice in the oil and gas investment decision-making field.

### Weight calculation

This study cornerstone is constructing a robust evaluation metric system and precisely quantifying each metric's weightage. Analytic Hierarchy Process (AHP) serves as a multi-criteria decision-making approach that facilitates the decomposition of complex issues into more manageable components by constructing a hierarchical model. This method is particularly effective for establishing the relative importance between hierarchical levels through a series of pairwise comparisons^31,33^. Herein, AHP is used to prioritize the subjective criteria layer and to determine the top-level weightage for the composite potential index system of petroleum resources. The key point hinges on the incorporation of subjective elements, specifically the expertise and industry experience of a panel of experts with relevant domain knowledge. Through iterative discussions and analyses, these experts collaboratively establish the intra-layer priority sequence for all three layers of indicators, as well as contribute to the definition of evaluation criteria and hierarchical structure.

The Entropy Weight Method is an objective weight-determination technique grounded in Information Theory^34–36^. This method capitalizes on the intrinsic information contained within raw data to minimize the interference of subjective judgments. It accomplishes this by analyzing the dispersion or uncertainty associated with each indicator to ascertain its weight. By integrating objective factors into the subjective elements, this method renders the weight allocation process more rational and scientifically robust. Within the framework of our model, the Entropy Weight Method is to determine the weightage of the middle layer of the indicator system, thereby ensuring both objectivity and accuracy in the evaluation process.

The Analytic Hierarchy Process (AHP) leverages the insights and experience of experts to establish subjective weightings, while the Entropy Weight Method contributes to the determination of objective weightings. This dual approach ensures the rationality of the evaluation, making the entire assessment process both comprehensive and precise. Sole reliance on either subjective or objective evaluation methods could result in biases stemming from expert preferences or the neglect of valuable experiential insights. The granularity of our indicators incorporates both subjective and objective elements. This amalgamation not only enriches the model with the invaluable expertise of professionals but also objectifies the complexity and scientific rigor of the process. Such a synergistic integration accentuates the unique advantages of our model.

Specific steps for calculating composite weight indicators through the integration of both methods are below:

Step 1: Construct a hierarchical model and judgment matrix.
  - Hierarchical model: The model comprises three layers: the Objective Layer (L1), the Criteria Layer (L2), and the Alternative Layer (L3).
  - Judgment matrix: Use Saaty 1–9 scale method to construct the judgment matrix $A=({a}_{ij}{)}_{n\times n}$.
Step 2: Perform hierarchical single sorting and consistency testing to determine the subjective weights in the analytic hierarchy process.
  - Data Normalization and Constructing the Normalized Judgment Matrix: ${a}_{ij}=\frac{{a}_{ij}}{\sum_{j=1}^{n} {a}_{ij}},$ where $i,j=\mathrm{1,2},\dots ,n$.
  - Weight Vector: Compute the average sum of the elements in each row of matrix A, i.e., ${w}_{i}=\frac{1}{n}\sum_{j=1}^{n} {a}_{ij}$, , where $[{w}_{1}\dots {w}_{n}{]}^{T}$, is the desired eigenvector.
  - Maximum Eigenvalue: Calculate ${\lambda }_{{\text{max}}}=\frac{1}{n}\sum_{i=1}^{n} \frac{(A\omega {)}_{i}}{{\omega }_{i}}$.
  - Consistency Index: the consistency index of the judgment matrix as $CI=\frac{{\lambda }_{{\text{max}}}-n}{n-1}$.
  - Calculate the Consistency Ratio: Set the Consistency Ratio as $CR=\frac{CI}{RI}$, where is the average random consistency index of the judgment matrix. A smaller CR indicates better consistency of the matrix.
Step 3: Perform data standardization and calculate the objective weights of information entropy.
  - Data standardization: Construct the assessment matrix $Y=({y}_{ij}{)}_{n\times m}$ and normalize it to ${E}_{ij}=\frac{{y}_{ij}}{\sum_{j=1}^{m} {y}_{ij}}$.
  - Information entropy: The value of indicator i is $H\left(i\right)=-\frac{1}{{\text{ln}}m}\sum_{j=1}^{m} {E}_{ij}{\text{ln}}{E}_{ij}$
  - Entropy weight for each indicator: The entropy weight of indicator i can be expressed as ${w}_{i}^{ }=\frac{1-H\left(i\right)}{\sum_{i=1}^{n} \left(1-H\left(i\right)\right)}$. The final vector of indicator weights obtained through the entropy weight method is ${W}_{i}=\left({w}_{1}^{ },\dots ,{w}_{i}\right)$.
Step 4: Calculate composite weights.
  - Combine subjective and objective weight information from Steps 2 and 3. The calculation formula for composite weights is
  - ${W}_{j}=\left(\frac{{\alpha }_{j}{\beta }_{j}}{\sum_{j=1}^{n} \sqrt{{\alpha }_{j}{\beta }_{j}}}\right)$where ${\alpha }_{j}$ and ${\beta }_{j}$ are the subjective and objective weights respectively, and ${W}_{j}$ is the composite weight factor.

Through these steps, this hybrid model allows the combination of subjective and objective assessments to analyze and evaluate complex issues in a consistent and comprehensive manner.

### Evaluation model construction

Incorporating the principles of fuzzy evaluation, the model utilizes fuzzy relational calculus to quantify factors that are traditionally non-quantifiable^37^. The Technique for Order of Preference by Similarity to Ideal Solution (TOPSIS), a multi-criteria decision-making method grounded in distance metrics^38^. TOPSIS employs the distance to both the ideal and anti-ideal solutions as criteria for ranking alternative scenarios, thereby revealing their relative merits and demerits^39,40^. Conventional TOPSIS methodologies often require a substantial volume of baseline data, with the assessment objective being that the closer an alternative is to the ideal solution, the more likely it is to approach the anti-ideal solution. To address this limitation, the present study employs an enhanced Grey Relational Analysis (GRA) to consolidate the foundational data. Subsequent computational steps are executed once the evaluative indicators within the framework of Structural Equation Modeling is definitively established.

Step 1: Weighted standardized evaluation matrix.
  The Z-score, also known as the standard score, serves as a statistical measure that describes the distance of an observed value from the mean of the entire dataset, expressed in units of standard deviation^41^. We transform the initial dataset into Z-scores, which are values that adhere to a standard distribution with a mean of zero and a standard deviation of one compared to the raw data. This normalization facilitates more straightforward comparisons across multiple datasets. Standardization is carried out using the Z-score method.
  1 $${q}_{ij}=\frac{{a}_{ij}-{\mu }_{i}}{{\sigma }_{i}}$$
  Then, calculate the weighted and normalized assessment matrix $Q=({q}_{ij}{)}_{p\times q}$, where ${q}_{ij}={q}_{ij}\cdot {v}_{i}$ and the weights are determined using a specific weight allocation method:
  - ${a}_{ij}$ is an element in the original assessment matrix.
  - ${\mu }_{i}$ is the mean of the column i.
  - ${\sigma }_{j}$ is the standard deviation of the j column.
Step 2: Positive and negative ideal solutions in exponential form
  The exponential calculation method will be used to highlight key or significant indicators in the positive and negative ideal solutions more explicitly, aiming to facilitate more accurate subsequent analysis.
  1. Ideal Positive Solution Q+: For each indicator, select the optimal value from all the alternative options. If the c indicator is of the "the larger, the better" type, choose the maximum value; if it is of the "the smaller, the better" type, opt for the minimum value. Combine these values into a vector to obtain the Ideal Positive Solution.
    2 $${\varvec{Q}}+=({\text{maxqi}}1,{\text{maxqi}}2,\dots ,{\text{maxqiq}})\mathrm{ \,or \,}({\text{minqi}}1,{\text{minqi}}2,\dots ,{\text{minqiq}})$$
    where the selection of either the maximum or minimum depends on the nature of the criteria.
  2. Ideal Positive Solution Q-: In contrast to the Ideal Positive Solution, the Ideal Negative Solution selects the least desirable value for each criterion. For criteria of the "the larger, the better" type, choose the minimum value; for those of the "the smaller, the better" type, opt for the maximum value. Combine these values into a vector to obtain the Ideal Negative Solution.
    3 $${\varvec{Q}}-=({\text{minqi}}1,{\text{minqi}}2,\dots ,{\text{minqiq}})\mathrm{ \,or \,}({\text{maxqi}}1,{\text{maxqi}}2,\dots ,{\text{maxqiq}})$$
    Similarly, the choice of maximum or minimum depends on the nature of the criteria.
Step 3: Grey relational matrix using the improved grey relational formula.
  4 $${g}_{ij}=\frac{\beta +\mathrm{min }\mid {q}_{i}^{+}-q{ }_{ij}\mid }{\beta +\mid {q}_{i}^{+}-q{ }_{ij}\mid }$$
  where β is the grey relational resolution coefficient, a positive constant less than 1. It is used to adjust the calculation of grey relational degree, enhancing computational stability and sensitivity. Here, we set it to 0.5.
Step 4: Grey relational coefficient matrices for the positive and negative ideal solutions using different methods.
  5 $$\begin{array}{cc}{G}^{+}& ={\text{max}}{g}_{ij}\mid l=\mathrm{1,2},\dots ,p={g}_{1}^{+},{g}_{2}^{+},\dots ,{g}_{p}^{+}\\ {G}^{-}& ={\text{min}}{g}_{ij}\mid l=\mathrm{1,2},\dots ,p={g}_{1}^{-},{g}_{2}^{-},\dots ,{g}_{p}^{-}\end{array}$$

## 3D spatial mapping

To optimize the classification and assessment of petroleum investment potential, we utilize a three-dimensional spatial analysis technique. By switching between various viewpoints, this approach facilitates the most straightforward visualization of the results we aim to achieve, grounded in our evaluative modeling studies. The coordinates for the basin can be ascertained using the following equations:

1. Three key indicators: Exploration Status (X), Development and Production Status (Y) and Local Environmental Conditions (Z)
2. Mapped onto the XYZ axes in three-dimensional space:
  Step 1: The coordinates of the basin are determined by the following formula:
    6 $$\begin{aligned} {x}_{i} & ={f}_{1}\left({\text{X}}_{i}\right) \\ {y}_{i} & ={f}_{2}\left({\text{Y}}_{i}\right) \\ {z}_{i} & ={f}_{3}\left({\text{Z}}_{i}\right) \end{aligned}$$
    where ${\text{X}}_{i} , {\text{Y}}_{i} and {\text{Z}}_{i}$ respectively represent indicators of Exploration Status, Development and Production and Local Environment.
  Step 2: Map the original data of transformation functions (${{\varvec{f}}}_{1}$, ${{\varvec{f}}}_{2}$, ${{\varvec{f}}}_{3}$) to appropriate scales and ranges.

### Standard deviation

Classifying data based on Standard Deviation is a commonly employed statistical technique for identifying the variability or dispersion within a dataset^42–44^. This method is frequently used for categorizing or segmenting data to facilitate easier analysis and interpretation. For a one-dimensional dataset X, with a mean μ and standard deviation σ, classification can be conducted using the following equations:

7 $$Classification(x)= \left \{\begin{array}{lll}\text{``}Poor\text{''}& \quad if \; x < \mu -\sigma, & \\ \text{``}Medium\text{''} & \quad if \; \mu -\sigma \le x\le \mu +\sigma, & \\ \text{``}Good\text{''}& \quad if \; x>\mu +\sigma . \end{array} \right.$$

The Lower Bound is defined as $\mu -\sigma$, while the Upper Bound is $\mu +\sigma$.

In the analysis, we operate under the assumption that higher values within dataset X correspond to better evaluations of CIPP. Consequently, we categorize the data into three classes: "Poor" (below one standard deviation from the mean), "Medium" (within one standard deviation of the mean, either above or below), and "Good" (above one standard deviation from the mean). This classification aims for a visualization that intuitively reflects the distribution of all basins according to CIPP values across three macro-dimensions of focus: Exploration Status, Development and Production and Local Environment. Under any two dimensions of potential interest, we can categorize basins into these three tiers based on calculated results. In a more flexible three-dimensional visualization, we can quickly and selectively identify basins with the characteristics we need.

### Spearman rank correlation analysis

Spearman rank correlation analysis serves as a nonparametric statistical approach for assessing the strength and directionality of the relationship between two variables^45,46^. This method is particularly well-suited for ordinal data or datasets that do not conform to the assumptions of a normal distribution. It offers a robust framework for evaluating correlations in nonlinear relationships, especially when the data are significantly influenced by outliers. The procedure for implementing this method is outlined as follows:

1. Data preparation: Ensure two sets of one-dimensional data for comparison: $X=\{{x}_{1},{x}_{2},\dots ,{x}_{n}\} and Y=\{{y}_{1},{y}_{2},\dots ,{y}_{n}\}$;
2. Data sorting and rank assignment: Sort the two sets of data individually and assign ranks to each data point within their respective datasets. Assign them an average rank when encountering tied values.
3. Calculating differences: For each pair ($x$,$y$), calculate the rank difference $d={\text{rank}}(x)-{\text{rank}}(y)$.
4. Spearman rank correlation coefficient $\rho$:
  8 $$\rho =1-\frac{6\sum {d}^{2}}{n({n}^{2}-1)}$$
  where $\sum {d}^{2}$ is the sum of the squares of all rank differences, and $n$ is the number of observations.
5. Result analysis: When the value of ρ is − 1 or 1, it indicates a perfect positive or negative correlation between the two variables. When the value of ρ is close to zero, the two variables are nearly uncorrelated. When 0 < ρ < 1, it represents a positive correlation, and when − 1 < ρ < 0, it represents a negative correlation. The value of ρ helps determine the strength and direction of the correlation between the two sets of data.

## Results

### Establish hierarchical indicator system

The task of identifying and deconstructing critical factors that influence the potential of petroleum resources holds crucial importance. We carefully deconstructed and ranked these factors, culminating in a final framework.

**Figure 2:**
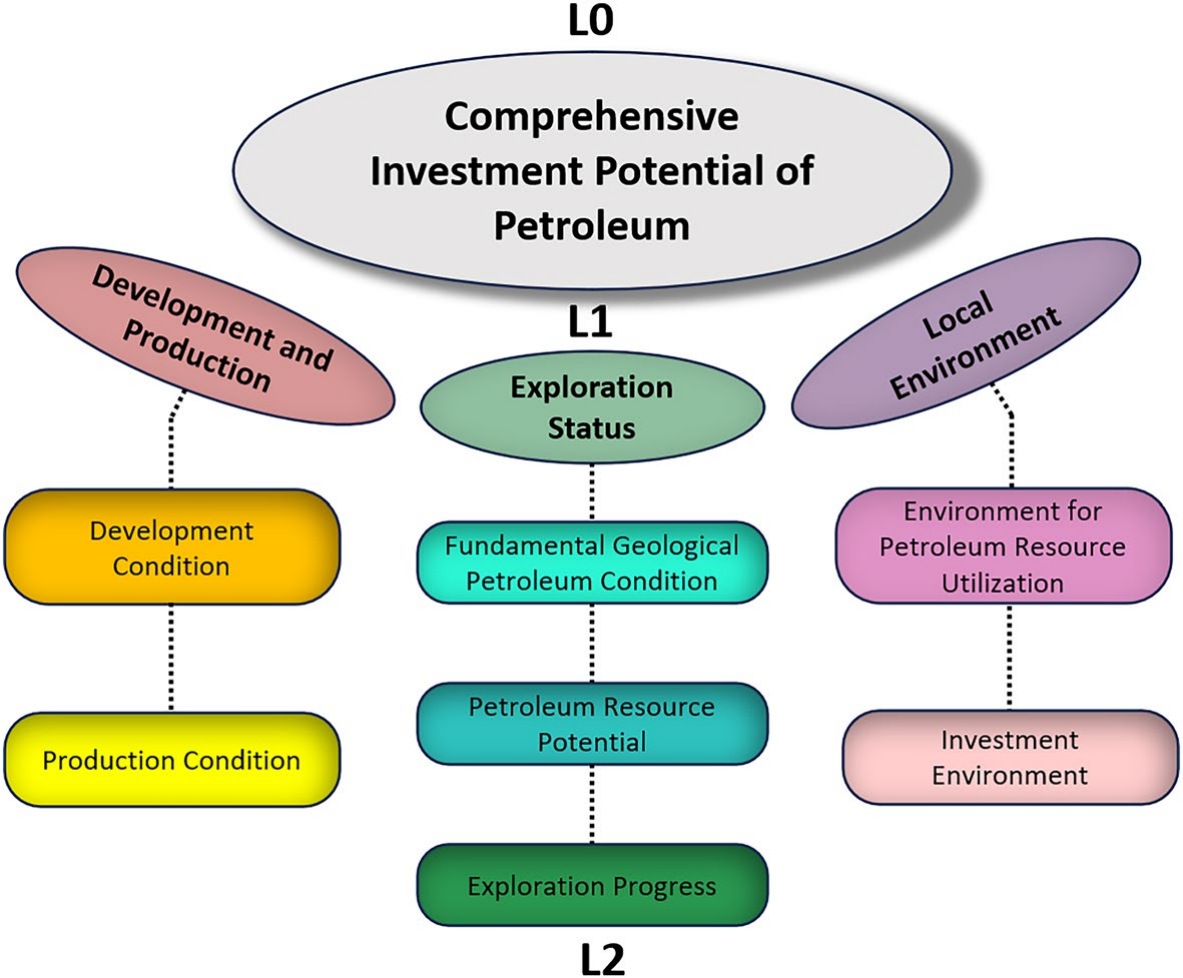
Three-layer decomposition schematic of the evaluation system for CIPP.

Factors affecting the potential of petroleum resources appear in our deconstructive model as three distinct tiers (Fig. 2). The first tier (L1) primarily encompasses foundational elements such as exploration status, resource potential, exploration progress, development and production, and local environmental conditions. These elements serve as the foundation for petroleum resource potential. The second tier (L2) delves into more specific intermediate factors like basic petroleum geological conditions, proven reserves, well density, development conditions, production conditions, resource utilization environment, and investment climate. These factors exert an indirect influence based on the foundational elements. The third tier (L3) further refines the factors from the second tier, including 32 high-level strategic elements such as source rock conditions, reservoir conditions, cap rock, and preservation conditions (Table 1). These elements wield decisive influence over the ultimate realization of petroleum resource potential.

This three-tiered analysis enables a comprehensive understanding of the complex structure and interrelationships affecting the potential for petroleum exploration. It offers robust support for further strategic planning and decision-making. The factors in each tier have been carefully selected and categorized through iterative discussions among the expert panel to ensure a holistic reflection of the multifaceted influences on petroleum resource potential. This integrated analytical approach highlights the interplay among multiple tiers of factors, revealing a complex system of influences on petroleum resource potential and providing robust theoretical support for a deeper understanding of the subject.

### Develop indicator weights

To quantify the progress of various petroleum basins in terms of investable development, we adopted a series of explicit evaluation criteria and data collection methods. The potential among different petroleum basins in Africa exhibits significant disparities. Notably, basins along the East and West coasts demonstrate higher investable development potential. For this analysis, we specifically selected 17 highly representative petroleum basins as subjects of study and utilized the most recent data from the year 2020. We applied data analysis using Python 3.0 (numpy, pandas, matplotlib etc.) to determine the weightings of various indicators. This weighting system integrates both subjective and objective factors to more comprehensively assess the performance potential of each petroleum basin.

The analysis results indicate that in the first-tier indicators (L1), CIPP primarily reflects investment potential from multiple angles, focusing on three aspects: Exploration Status, Development and Production, and Local Environment. Particularly for Exploration Status, due to its reflection of petroleum reserves and development potential, it received appropriate emphasis in the weight distribution. In the second-tier indicators, the impact of Exploration Progress is most significant, with a weight of 0.314. This underscores the crucial role that petroleum reserves and exploration clarity play in investment funding. The weight distribution for other indicators is detailed in the table. Through the structured three-tier model based on CIPP, a clear combination of subjective and objective weight distribution provides a comprehensive and quantitative assessment method for considering the strategic level of transnational overseas petroleum investments.

### Evaluation results

**Figure 3:**
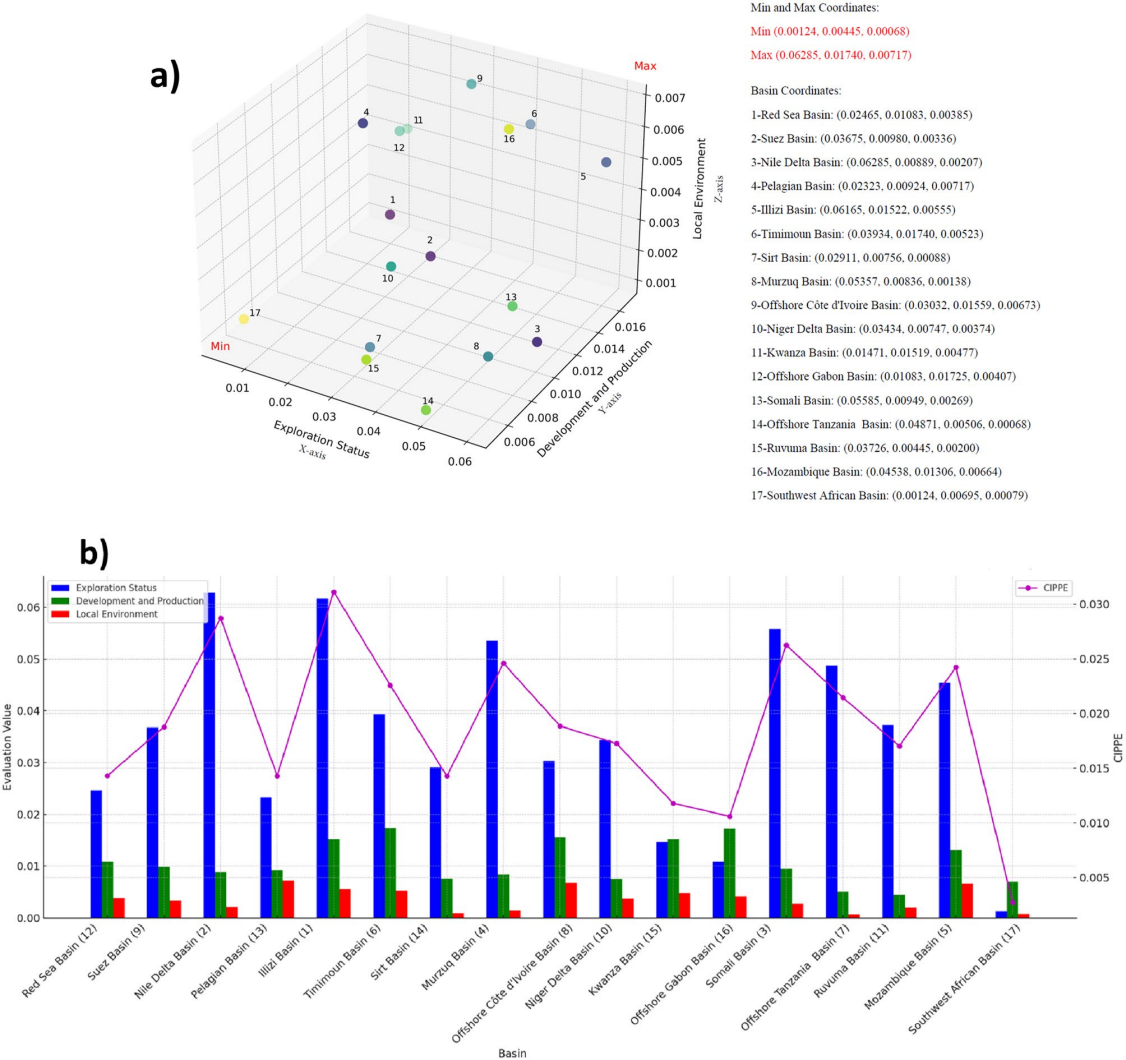
Evaluation results of 17 representative petroleum basins: (**a**) three-dimensional plotting and ranking based on three L1 indicators as axes. (**b**) Bar chart of the assessment values for all basins, indicating one L0 and three L1 assessment values.

After the model analysis, we calculated the composite evaluation results for 17 key and representative basins (Table 2 and Fig. 3). We quantified various aspects of the petroleum development potential of these basins through a specific assessment model, including their strengths and weaknesses. This data reveals the comprehensive investment potential in petroleum resources for each basin, as well as their potential values in Exploration Status, Development and Production, and Local Environment.

**Table 2:** Evaluation values for 17 representative petroleum basins derived through complex model processing across levels of L0, L1 and L2.

No.	Basin	L0	L1			L2						
		CIPP	Exploration status	Development and production	Local environment	Fundamental geological petroleum condition	Petroleum resource potential	Exploration progress	Development condition	Production condition	Environment for petroleum resource utilization	Investment environment
1	Illizi Basin	0.03113	0.06165	0.01522	0.00555	0.05283	0.13354	0.06391	0.09059	0.06825	0.06069	0.03228
2	Nile Delta Basin	0.02873	0.06285	0.00889	0.00207	0.01762	0.14255	0.06731	0.03272	0.04608	0.02752	0.00854
3	Somali Basin	0.02625	0.055854	0.009489	0.002687	0.01880	0.14457	0.04295	0.02681	0.05164	0.02427	0.01930
4	Murzuq Basin	0.02460	0.05357	0.00836	0.00138	0.00861	0.14643	0.03622	0.01342	0.04864	0.01543	0.00785
5	Mozambique Basin	0.02423	0.04538	0.01306	0.00664	0.05067	0.09899	0.04380	0.06128	0.06358	0.04843	0.05589
6	Timimoun Basin	0.02258	0.03934	0.01740	0.00523	0.06413	0.02640	0.08733	0.05378	0.09329	0.07093	0.02066
7	Offshore Tanzania Basin	0.02146	0.04871	0.00506	0.00068	0.01133	0.13169	0.03347	0.01906	0.02608	0.00667	0.00454
8	Offshore Côte d'Ivoire Basin	0.01886	0.03032	0.01559	0.00673	0.08043	0.00631	0.07319	0.06929	0.07708	0.04006	0.06313
9	Suez Basin	0.01878	0.03675	0.00980	0.00336	0.01154	0.04990	0.06937	0.03814	0.05012	0.03530	0.02059
10	Niger Delta Basin	0.01725	0.03434	0.007474	0.003738	0.02329	0.07082	0.04017	0.05101	0.03151	0.03040	0.02924
11	Ruvuma Basin	0.01704	0.03726	0.00445	0.00200	0.01213	0.11505	0.01188	0.04340	0.01476	0.01976	0.01308
12	Red Sea Basin	0.01431	0.02465	0.01083	0.00385	0.01391	0.00825	0.06801	0.07468	0.04545	0.02784	0.03260
13	Pelagian Basin	0.01429	0.02323	0.00924	0.00717	0.04445	0.00711	0.05778	0.06628	0.03797	0.04947	0.06243
14	Sirt Basin	0.01427	0.02911	0.00756	0.00088	0.00299	0.10001	0.00157	0.03224	0.03778	0.01428	0.00182
15	Kwanza Basin	0.01179	0.01471	0.01519	0.00477	0.03481	0.00650	0.03331	0.12697	0.05689	0.03678	0.03869
16	Offshore Gabon Basin	0.01059	0.01083	0.01725	0.00407	0.02063	0.00439	0.02599	0.04210	0.09594	0.03944	0.02725
17	Southwest African Basin	0.00273	0.00124	0.00695	0.00079	0.00092	0.00038	0.00340	0.03875	0.03197	0.01175	0.00245

In the comprehensive analysis of petroleum basin investment potential in this study, we applied an in-depth quantitative assessment using two main discount indicators, i.e., L0 and L1, along with three sub-indicators (Exploration Status, Development and Production, Local Environment). The three-dimensional visualization (Fig. 3a) better illustrates the distribution of these basins at the L0 level. It is evident that the point aggregation is very low, indicating significant variability among the three indicators. From the overall trend analysis (Fig. 3b), the disparity among the three indicators across different basins is particularly large, leading to a significant difference in their overall potential. Specifically, the "llizi Basin" and "Nile Delta Basin" have L0 and L1 significantly outperform other basins and demonstrate their strong investment potential. However, the "Southwest African Basin" and "Offshore Gabon Basin" rank last, suggesting their lower investment value potential. On the L0 and L1 indicators, the "llizi Basin" and "Nile Delta Basin" performed best, while the "Southwest African Basin" and "Offshore Gabon Basin" scored the lowest. At the L1 level, the "Timimoun Basin" and "Offshore Gabon Basin" performed best in Exploration Status. The "Pelagian Basin" and "Offshore Cote d'Ivoire Basin" scored highest in Development and Production and Local Environment.

### Classification

We further classified the basins, initially classifying them based on three indicators. Utilizing the classification method based on Standard Deviation, we divided each basin into four levels across these three distinct indicators. These four levels respectively represent excellence in all three indicators, followed by the next two, one, and none.

**Figure 4:**
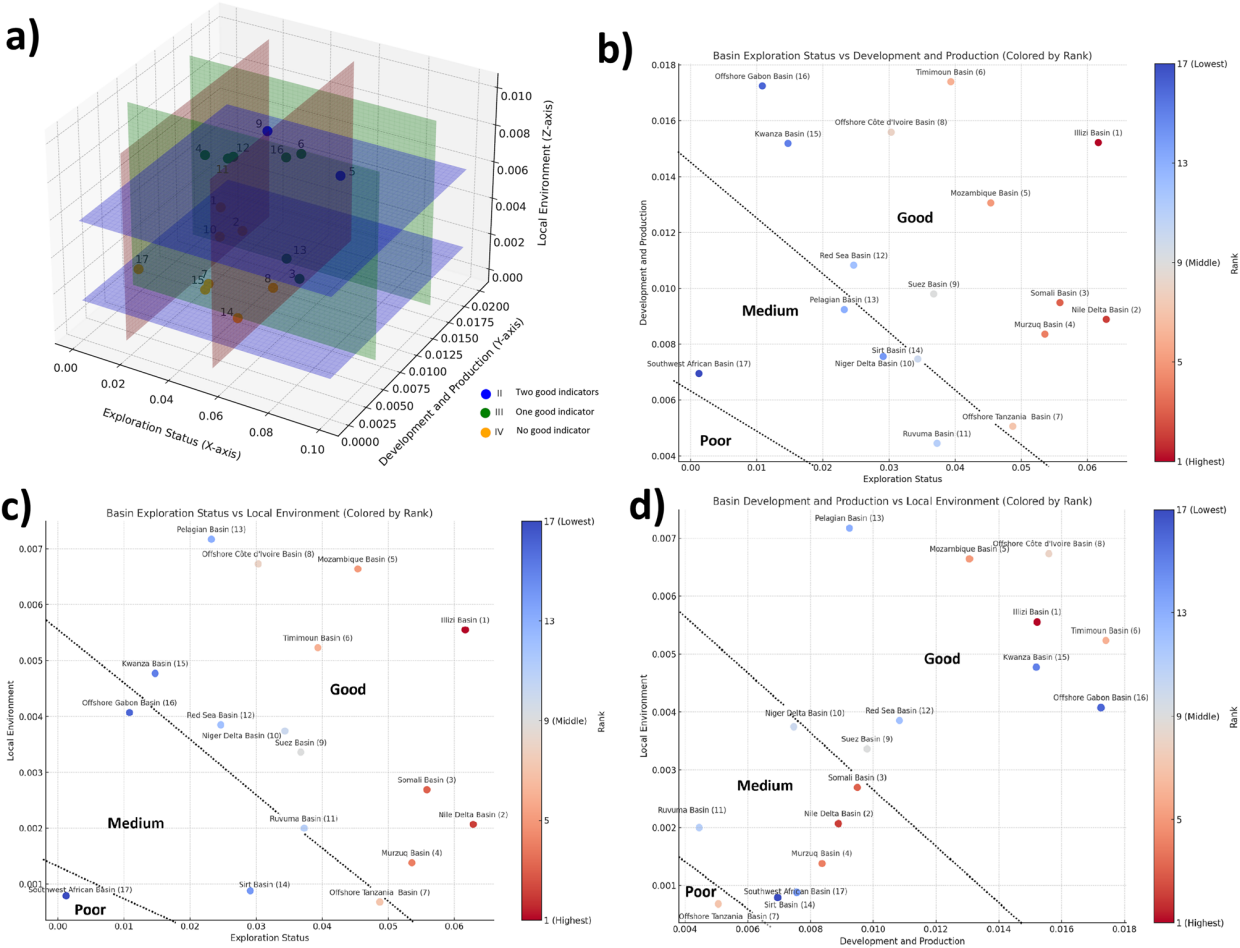
Classification results: (**a**) classification on the 3D plot based on the analysis of evaluation values and subsequent classification using the standard deviation model. (**b**) Basin classification on the axes of exploration status vs development and production. (**c**) Basin classification on the axes of exploration status vs development and production and local environment. (**d**) Basin classification on the axes of development and production and local environment.

The classification results indicate that for "Exploration Status," both the "Nile Delta Basin" and "llizi Basin" are rated as "Good," while the "Kwanza Basin," "Offshore Gabon Basin," and "Southwest African Basin" are categorized as "Poor" (Fig. 4). In terms of "Development and Production," the "llizi Basin," "Timimoun Basin," "Offshore Cote d'Ivoire Basin," "Kwanza Basin," and "Offshore Gabon Basin" received "Good" ratings. As for "Local Environment," the "Pelagian Basin," "Offshore Cote d'Ivoire Basin," and "Mozambique Basin" performed best, in contrast to the "Sirt Basin," "Murzug Basin," "Niger Delta Basin," "Offshore Tanzania Basin," and "Southwest African Basin," which showed poorer performance on this indicator.

The CIPP evaluation model framework provides a multi-tiered classification method, allowing petroleum investors and decision-makers to more accurately identify various investment opportunities and risks at the basin level. Since none of the selected representative investable basins fall into Type I, the classification for Categories II to IV is as follows:

1. Type II Basins (Illizi Basin and Offshore Côte d'Ivoire Basin): These basins typically exhibit significant advantages in resource exploration and development production. In such cases, investors may consider these basins as medium-to-long-term investment targets. Due to their strong performance in two key indicators, these basins have a higher probability of success and potential returns. However, as there is room for improvement in the third aspect (usually local environmental adaptation), investors may need additional environmental and social responsibility plans to ensure the sustainability of the projects.
2. Type III Basins (Nile Delta Basin, Pelagian Basin, Timimoun Basin, Kwanza Basin, Offshore Gabon Basin, Somali Basin and Mozambique Basin): These basins exhibit higher levels of risk and uncertainty. They typically excel in only one aspect while performing moderately in the other two. This implies that investors should exercise greater caution in their investment decisions and may require additional research and risk mitigation measures. For instance, if a Type III basin shows strong performance in resource exploration but is mediocre in development production and environmental adaptability, investors may need to allocate additional technical and capital resources to improve these two aspects.
3. Type IV Basins (Red Sea Basin, Suez Basin, Sirt Basin, Murzuq Basin, Niger Delta Basin, Offshore Tanzania Basin, Ruvuma Basin and Southwest African Basin): These are generally not ideal investment targets. These basins perform poorly across all considered factors and typically require comprehensive strategic adjustments, including improvements in exploration techniques, optimization of production processes, and enhanced environmental protection measures. Therefore, unless there are compelling reasons and preparations for large-scale reforms and investments, these basins should generally be avoided for investment.

### Correlation analysis

**Figure 5:**
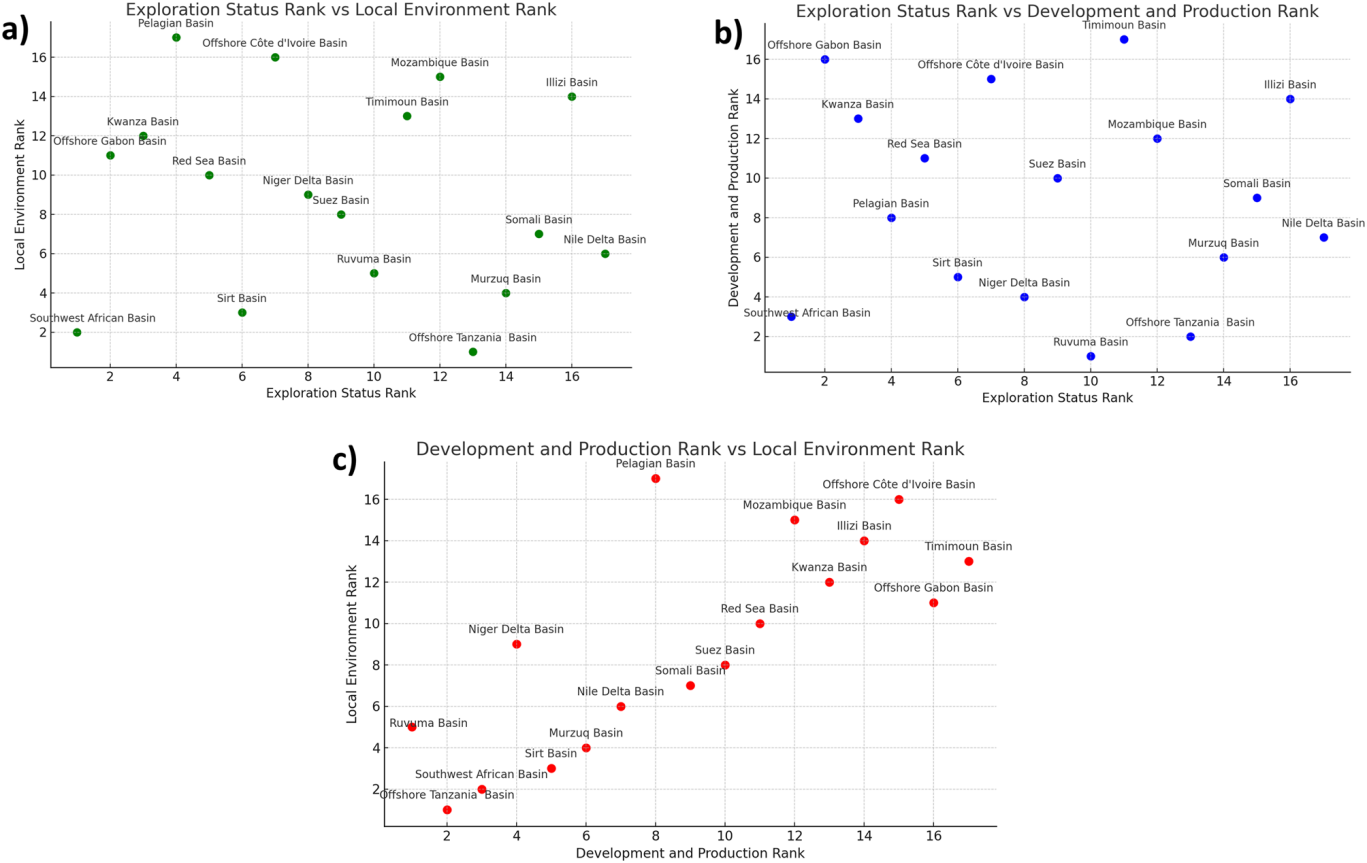
Correlation analysis with any two of the three indicators as axes: (**a**) exploration status vs development and local environment and (**b**) exploration status vs development and production, both show low correlation due to high dispersion. (**c**) Development and production vs local environment shows a certain level of correlation.

Conducting a Spearman correlation analysis between these three key indicators can be crucial for understanding their interrelationships and optimizing resource management and policy decisions. Based on Fig. 5a, the correlation between "Exploration Status" and "Local Environment" is relatively weak. Figure 5b reveals a moderate positive correlation between "Exploration Status" and "Development and Production". Importantly, this result is highly statistically significant, with a p-value of merely 0.001. This suggests that basins ranking high in Exploration Status often also rank high in Development and Production. Lastly, according to Fig. 5c, a moderate positive correlation exists between "Development and Production" and "Local Environment". This implies that basins selected for development and production often also perform well in local environmental aspects, although this positive correlation is not strong. Overall, these findings suggest a notable positive correlation between Exploration Status and Development and Production, while the association with Local Environment is comparatively weaker.

## Discussion

This study produces an evaluation system based on an Interpretive Structural Model to form the CIPP to assess comprehensive petroleum investment potential. This workflow integrates factors from petroleum geology as well as political, economic and cultural dimensions, adapting complex models commonly used in other fields for this assessment. In doing so, it fills a research gap in the field of transnational petroleum investment at basin level, elevating the focus beyond the primarily economic aspects that have dominated past research in this area (e.g.,^11,12,15,30^).

The study primarily innovates by quantitatively assessing 17 representative African oil basins through an integrated evaluation model, aiming to identify and compare the differences among these basins in terms of oil resource exploration, development potential, and their investment attractiveness. By applying the Analytic Hierarchy Process (AHP), Entropy Weight Method, and Fuzzy TOPSIS technique, the research constructs a comprehensive evaluation system to quantitatively analyze each CIPP of each basin in the three aspects of Exploration Status, Development and Production and Local Environment.

The results suggest that the Illizi Basin and Offshore Côte d'Ivoire Basin are highly favorable for investment and development in petroleum resource exploration, particularly for various enterprises including those from China. This attractiveness is not solely based on abundant petroleum resources and favorable infrastructure, but also takes into account political and economic stability. However, basins such as the Offshore Tanzania Basin, Ruvuma Basin and Southwest African Basin are assessed as options that require very cautious consideration for investment. They rank low on a comprehensive level, and their limitations are not confined to just one aspect. This approach can assist Chinese or other oil and gas companies in gaining a clearer understanding at the basin level when investing in upstream oil and gas in Africa.

The optimal outcome of this ranking aligns closely with our professional opinion, which is derived from extensive experience and based on thorough investigation and analysis of a significant amount of expert outcomes. We believe that the Illizi Basin and Offshore Côte d'Ivoire Basin are the most favorable regions at the basin level for investment and development by Chinese oil and gas enterprises, based on our comprehensive assessment. These basins have been in a state of global oil and gas investment for some time, and they have relatively well-developed infrastructure and regulatory frameworks. With effort, we can successfully secure a share of the benefits. However, regarding the Tanzania Basin, Ruvuma Basin, and Southwest African Basin, although they may have considerable oil and gas reserves, the local development environment is relatively harsh, and the political risks and investment climate are also a matter for concern. Therefore, the model analysis fits our manual interpretation with a highly cautious approach to these regions.

This study categorizes 17 African oil basins into four levels based on Exploration Status, Development and Production, and Local Environment, using the standard deviation method. This classification aids investors and decision-makers in pinpointing specific investment opportunities and assessing potential risks. Basins are ranked from those excelling in all three indicators (Type II), indicating highest investment value, to those needing improvement (Type III), and those performing poorly (Type IV), reflecting varying investment appeal and development potential. Moreover, Spearman rank correlation analysis shows a moderate positive correlation between Exploration Status and Development and Production, with a weaker correlation to Local Environment. This highlights the importance of exploration status as a predictive indicator for a basin development potential but also underscores the necessity of considering a wide range of factors for comprehensive investment decisions. This approach assists in more precise resource allocation and risk management for oil and gas investments in Africa.

This study enhances the ranking of oil and gas exploration potential across various African basins and includes detailed dimensional rankings as well as comparisons between dimensions of the same level. This aspect, even for global petroleum investment field, was absent in previous studies^8–10^, which were primarily limited to the financial investment dimension^16^, lacking consideration of the oil and gas geological aspects of basins and downstream infrastructure levels. Furthermore, complex decision-making models have rarely been applied to such oil and gas investments before^23,24^ . Our research, while differing slightly in analysis details and incorporating a greater number of parameters for evaluation and classification compared to similar algorithms in other domains, effectively aids in prioritizing decision analysis objectives related to complexity^27,28^. The application of this model provides a very good example of integration with actual investment behaviors, serving as a reference for similar decision-making fields or inspiring research on similar model applications.

This approach enhances decision accuracy, addresses uncertainty and yields clear, interpretable results, ultimately facilitating more effective decision-making. This advanced approach contributes to the algorithm advancement by handling complexity, enhances its transferability to various contexts, as well as improves its applicability in decision-making across industries and regions. More importantly, we can evaluate the ranking of each basin in-depth based on different sub-indicators and then determine the priority situation.

This modeling methodology focuses on assessing the petroleum investment potential of oil and gas basins, leveraging the practical experience of Chinese enterprises in overseas investments, specifically tailored for Africa due to its unique complexity in the mentioned aspects. Cross-regional comparisons, such as with basins in the Middle East and South America, are not considered within the framework of this model. For evaluations of specific basin groups, indicators with high commonality could be excluded in favor of new indicators with greater heterogeneity, integrating human interpretation with actual data to recalibrate weights. This model emphasizes the amalgamation of subjective and objective assessments in indicator ranking, innovatively influencing weight calculations to effectively integrate both evaluations comprehensively, departing from traditional segregations of subjective and objective metrics.

This evaluation system provides a practical application for a novel combined method for assessing petroleum basins in Africa. By classifying and quantitatively scoring these basins, petroleum investment companies can not only gain a more accurate understanding of the specific strengths and weaknesses of each basin but also systematically evaluate their potential risks and returns. The workflow is based on complex evaluation model methods that have already seen deep application in other fields^23–26^. This adaptation can be considered a significant contribution to the upstream petroleum resource investment.

Additionally, the classification and correlation work enrich the content of this comprehensive evaluation system. The results of the correlation study indicate that the three classification indicators, namely "Exploration Status," "Local Environment," and "Development and Production," exhibit certain interdependencies. Specifically, the first two show a moderate positive correlation, while the latter two are also related. This suggests that in petroleum basins with weak petroleum reserves but high exploration potential, there is already significant involvement from international petroleum companies, or the local government is likely to give it adequate attention.

While this study effectively evaluates petroleum basin investment potential, it also highlights limitations for future research to address. First, the current evaluation metrics need refinement to better capture the complexities of investment decisions, especially when considering multiple factors like economics and environment. Additional data, such as geological findings and market demand, should be included. Second, our model for assessing CIPP has limitations, notably in interpreting indicators such as exploration well density and success rate for example. High values do not always mean high potential, primarily due to deviations at extremely high or low data levels from the expectations of this study. Within a reasonable range (all indicators apply herein), these values demonstrate a proportional relationship, and we assume that no indicator experiences excessive conditions. Third, advanced data analytics like big data and AI should be used to improve the accuracy of investment and risk forecasts.

Lastly, robust model validation is essential to minimize biases and provide a stable basis for decision-making. Given the subjective elements involved in the evaluation process, there is a potential for bias in the presented results. However, it is important to note that our qualitative ranking of subjective interpretations relies on a thorough understanding of local contexts, strong domain expertise, and extensive discussions followed by multiple rounds of sorting and comparison to mitigate errors. To further mitigate bias, we propose several measures for future work. These include incorporating independent data sources for cross-validation, implementing expert blind review or expert validation panels, utilizing Monte Carlo simulation to address uncertainty, conducting sensitivity analyses, and validating results through comparative case studies, among other methods. These steps will enhance the reliability of investment strategies in the field of petroleum exploration and development.

This research aids China and other countries in deepening the analysis and planning of social policies related to African oil and gas investment decisions. Rationalizing Africa's oil and gas investments is crucial for enhancing energy security, advancing the Belt and Road Initiative, and strengthening Sino-African economic and political ties. Such investments not only boost China's diplomatic influence in Africa through deepened energy cooperation and solidifying friendly relations with African nations but also support China's global diplomatic policies. For African countries, rational oil and gas investments significantly spur local economic development and job creation, especially in oil and gas development and infrastructure. Moreover, improving infrastructure not only fosters African economic growth and enhances the living standards of the local population but also contributes to sustainable development goals. In the long term, Sino-African energy cooperation is expected to create a mutually beneficial situation, strengthening economic collaboration between both sides and promoting global energy market stability and development.

## Conclusion

This study applies a complex evaluation model to explore previously uncharted territories in transnational petroleum investment, focusing on target asset evaluation, classification and ranking. This work breaks new ground in terms of research scope, methodological transferability and model innovation. The key highlighted points of this work include:

- The results suggest that the Illizi Basin and Offshore Côte d'Ivoire Basin are highly favorable for investment and development in petroleum resource exploration, particularly for various enterprises including those from China. However, basins such as the Offshore Tanzania Basin, Ruvuma Basin and Southwest African Basin require very cautious consideration for investment due to their lower comprehensive potential.
- This study produces an evaluation system based on an Interpretive Structural Model to assess comprehensive petroleum investment potential. The integrated evaluation model combining AHP, entropy method and fuzzy TOPSIS provides an effective and practical application model to quantify the investment potential of complex petroleum basins.
- The combination of objective and subjective weighting methods renders the entire evaluation process more balanced. The classification approach reveals the relative strengths and weaknesses of different basin types, aiding investment decision-making.
- A moderate positive correlation exists between exploration status and development/production potential for the basins. The classification and correlation work enrich the content of this comprehensive evaluation system.
- Adaptation of complex evaluation models from other fields helps address the gap in comprehensive petroleum investment assessments.
- The workflow offers strategic insights and an advanced methodology for optimizing investment decisions across industries.
- This research supports China and other countries in enhancing social policy planning related to African oil and gas investments, crucial for energy security, the Belt and Road Initiative and Sino-African relations, fostering mutual economic growth, sustainable development and global energy stability.

## Data Availability

For reasons related to confidentiality, certain source code and interpreted data cannot be made publicly available in their entirety. If you require access, kindly reach out to the corresponding author via email. Selected important analysis data have been included in the related files.
